# 1-[(4-Bromo­phen­yl)(morpholin-4-yl)­meth­yl]naphthalen-2-ol

**DOI:** 10.1107/S160053681200459X

**Published:** 2012-02-24

**Authors:** Qun Zhao

**Affiliations:** a School of Pharmaceutical Sciences, Nanjing University of Chinese Medicine, Nanjing 210046, People’s Republic of China

## Abstract

The title compound, C_21_H_20_BrNO_2_, was obtained *via* a one-pot synthesis from the reaction of 4-bromo­benzaldehyde, 2-naphthol and morpholine. In the asymmetric unit, there are four mol­ecules with similar structures. The morpholine ring adopts a chair conformation, and the hy­droxy group links with the morpholine *via* an intra­molecular O—H⋯N hydrogen bond. The bromo­phenyl ring is approximately perpendicular to the mean pane of the naphthalene system at dihedral angles of 76.7 (3), 81.4 (3), 79.7 (3) and 84.5 (3)° in the four independent mol­ecules. Weak C—H⋯O hydrogen bonds are observed in the crystal.

## Related literature

For the application of the Betti-type reaction, see: Gardiner & Raston (1997 ▶); Gutsche & Nam (1998 ▶).
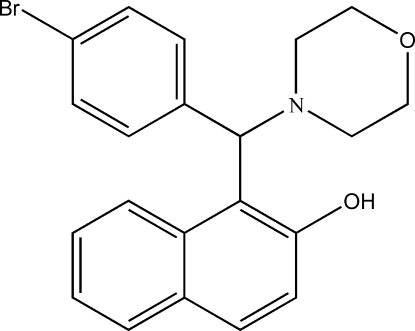


## Experimental

### 

#### Crystal data

C_21_H_20_BrNO_2_
*M* *_r_* = 398.28
Triclinic, 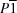

*a* = 10.514 (2) Å
*b* = 10.700 (2) Å
*c* = 34.002 (7) Å
α = 82.32 (3)°
β = 89.43 (3)°
γ = 88.67 (3)°
*V* = 3789.8 (13) Å^3^
*Z* = 8
Mo *K*α radiation
μ = 2.18 mm^−1^
*T* = 293 K
0.20 × 0.20 × 0.20 mm

#### Data collection

Rigaku Mercury2 (2 × 2 bin mode) diffractometer
Absorption correction: multi-scan (*CrystalClear*; Rigaku, 2005 ▶) *T*_min_ = 0.822, *T*_max_ = 1.000
26170 measured reflections
13194 independent reflections
5620 reflections with *I* > 2σ(*I*)
*R*_int_ = 0.094

#### Refinement

*R*[*F* ^2^ > 2σ(*F* ^2^)] = 0.083
*wR*(*F* ^2^) = 0.192
*S* = 1.03
13194 reflections
901 parameters
H-atom parameters constrained
Δρ_max_ = 0.59 e Å^−3^
Δρ_min_ = −0.58 e Å^−3^

### 

Data collection: *CrystalClear* (Rigaku, 2005 ▶); cell refinement: *CrystalClear*; data reduction: *CrystalClear*; program(s) used to solve structure: *SHELXS97* (Sheldrick, 2008 ▶); program(s) used to refine structure: *SHELXL97* (Sheldrick, 2008 ▶); molecular graphics: *SHELXTL* (Sheldrick, 2008 ▶); software used to prepare material for publication: *SHELXTL*.

## Supplementary Material

Crystal structure: contains datablock(s) I, global. DOI: 10.1107/S160053681200459X/xu5461sup1.cif

Structure factors: contains datablock(s) I. DOI: 10.1107/S160053681200459X/xu5461Isup2.hkl

Supplementary material file. DOI: 10.1107/S160053681200459X/xu5461Isup3.cml

Additional supplementary materials:  crystallographic information; 3D view; checkCIF report

## Figures and Tables

**Table 1:** Hydrogen-bond geometry (Å, °)

*D*—H⋯*A*	*D*—H	H⋯*A*	*D*⋯*A*	*D*—H⋯*A*
O1—H1*A*⋯N1	0.82	1.92	2.600 (7)	139
O3—H3*B*⋯N2	0.82	1.92	2.601 (7)	140
O5—H5*A*⋯N3	0.82	1.91	2.612 (7)	142
O7—H7*B*⋯N4	0.82	1.93	2.620 (7)	142
C25—H25*A*⋯O4^i^	0.93	2.58	3.473 (10)	162
C46—H46*A*⋯O6^ii^	0.93	2.57	3.371 (10)	144
C55—H55*A*⋯O5	0.93	2.59	3.305 (9)	134
C67—H67*A*⋯O8^iii^	0.93	2.41	3.208 (9)	143

Symmetry codes: (i) 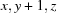; (ii) 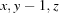; (iii) 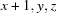.

**Fig. 1.**
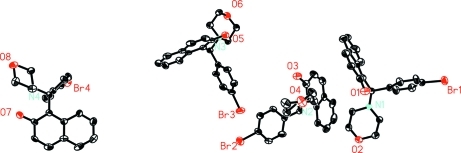
Perspective structure of the title compound, showing the atom-numbering scheme. Displacement ellipsoids are drawn at the 30% probability level.

**Fig. 2.**
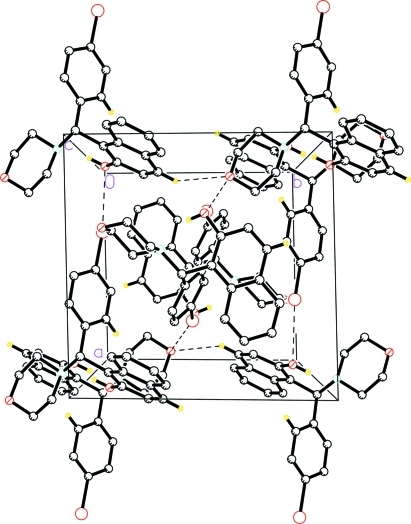
The crystal packing of the title compound viewed along thec axis showing the hydrogen bondings network.

## supplementary crystallographic information

### Comment

The reaction of substituted phenols and aldehydes under controlled conditions has been used to build up supramolecular compounds, the most important ones being calixarenes (Gardiner & Raston, 1997; Gutsche & Nam, 1998). 2-Naphthol reacts with aromatic aldehydes to produce 14-aryl-14*H*-dibenzo[a,j]xanthenes, which could be used as anti-inflamatory agents. Herein we report the synthesis and crystal structure of the title compound (Fig. 1).

In the title compound, bond lengths and angles have normal values. There are four molecules in the asymmetric unit, all having similar conformations. The morpholine rings adopt chair conformation. The dihedral angles between rings A (C1–C10) and B (C12–C17), and between rings C(C22–C31) and D(C33–C38), and between rings E(C43–C52) and F (C54–C59), and between rings G(C64–C73) and H(C75–C80), are 76.7 (3), 81.4 (3), 79.7 (3) and 84.5 (3)°, respectively. The crystal structure is stabilized by intermolecular C–H···O hydrogen bonds, which link the molecules into a three-dimensional network(Fig. 2). The intramolecular O–H···N hydrogen bonds are also present, Table 1.

### Experimental

4-Bromobenzaldehyde (2.76 g, 0.015 mol) and morpholine (1.305 g, 0.015 mol) was added to 2-naphthol (2.16 g, 0.015 mol) without solvent under nitrogen. The temperature was raised to 120°C in one hour gradually and the mixture was stirred at this temperature for 12 h. The system was treated with 30 ml of ethanol 95% and cooled. The precipitate was filtered and washed with a small amount of ethanol 95%. The title compound was isolated using column chromatography (Petroleum ether: ethyl acetate-5:1). Single crystals suitable for X-ray diffraction analysis were obtained from slow evaporation of a solution of the title compound in ethyl acetate at room temperature.

### Refinement

H atoms were positioned geometrically and refined using a riding model with C—H = 0.93–0.97 Å and O—H = 0.82 Å, U_iso_(H) = 1.2U_eq_(C) and 1.5U_eq_(O).

### Crystal data

**Table:**

C_21_H_20_BrNO_2_	*Z* = 8
*M**_r_* = 398.28	*F*(000) = 1632
Triclinic, *P*1	*D*_x_ = 1.396 Mg m^−^^3^
Hall symbol: -P 1	Mo *K*α radiation, λ = 0.71073 Å
*a* = 10.514 (2) Å	Cell parameters from 13194 reflections
*b* = 10.700 (2) Å	θ = 2.7–25.0°
*c* = 34.002 (7) Å	µ = 2.18 mm^−^^1^
α = 82.32 (3)°	*T* = 293 K
β = 89.43 (3)°	Prism, colorless
γ = 88.67 (3)°	0.20 × 0.20 × 0.20 mm
*V* = 3789.8 (13) Å^3^	

### Data collection

**Table:**

Rigaku Mercury2 (2x2 bin mode) diffractometer	13194 independent reflections
Radiation source: fine-focus sealed tube	5620 reflections with *I* > 2σ(*I*)
Graphite monochromator	*R*_int_ = 0.094
Detector resolution: 13.6612 pixels mm^-1^	θ_max_ = 25.0°, θ_min_ = 3.0°
CCD_Profile_fitting scans	*h* = −12→12
Absorption correction: multi-scan (*CrystalClear*; Rigaku, 2005)	*k* = −12→12
*T*_min_ = 0.822, *T*_max_ = 1.000	*l* = −40→40
26170 measured reflections	

### Refinement

**Table:**

Refinement on *F*^2^	Primary atom site location: structure-invariant direct methods
Least-squares matrix: full	Secondary atom site location: difference Fourier map
*R*[*F*^2^ > 2σ(*F*^2^)] = 0.083	Hydrogen site location: inferred from neighbouring sites
*wR*(*F*^2^) = 0.192	H-atom parameters constrained
*S* = 1.03	*w* = 1/[σ^2^(*F*_o_^2^) + (0.0538*P*)^2^ + 0.6976*P*] where *P* = (*F*_o_^2^ + 2*F*_c_^2^)/3
13194 reflections	(Δ/σ)_max_ = 0.004
901 parameters	Δρ_max_ = 0.59 e Å^−^^3^
0 restraints	Δρ_min_ = −0.58 e Å^−^^3^

### Special details

**Table:**

Geometry. All e.s.d.'s (except the e.s.d. in the dihedral angle between two l.s. planes) are estimated using the full covariance matrix. The cell e.s.d.'s are taken into account individually in the estimation of e.s.d.'s in distances, angles and torsion angles; correlations between e.s.d.'s in cell parameters are only used when they are defined by crystal symmetry. An approximate (isotropic) treatment of cell e.s.d.'s is used for estimating e.s.d.'s involving l.s. planes.
Refinement. Refinement of *F*^2^ against ALL reflections. The weighted *R*-factor *wR* and goodness of fit *S* are based on *F*^2^, conventional *R*-factors *R* are based on *F*, with *F* set to zero for negative *F*^2^. The threshold expression of *F*^2^ > σ(*F*^2^) is used only for calculating *R*-factors(gt) *etc*. and is not relevant to the choice of reflections for refinement. *R*-factors based on *F*^2^ are statistically about twice as large as those based on *F*, and *R*- factors based on ALL data will be even larger.

### Fractional atomic coordinates and isotropic or equivalent isotropic displacement parameters (Å^2^)

**Table:**

	*x*	*y*	*z*	*U*_iso_*/*U*_eq_	
Br1	0.54748 (9)	0.74290 (8)	1.00437 (3)	0.0785 (3)	
O1	0.4085 (5)	0.3444 (5)	0.83872 (16)	0.0707 (16)	
H1A	0.4775	0.3679	0.8456	0.106*	
O2	0.8290 (5)	0.3972 (5)	0.79797 (17)	0.0687 (15)	
N1	0.5990 (5)	0.4965 (5)	0.82932 (16)	0.0439 (15)	
C18	0.6225 (7)	0.4945 (7)	0.7866 (2)	0.052 (2)	
H18A	0.6599	0.5733	0.7752	0.062*	
H18B	0.5424	0.4863	0.7734	0.062*	
C1	0.3726 (7)	0.5702 (7)	0.8216 (2)	0.0446 (18)	
C12	0.5094 (7)	0.6366 (7)	0.8765 (2)	0.0492 (19)	
C2	0.3359 (8)	0.4461 (8)	0.8242 (2)	0.059 (2)	
C11	0.5067 (6)	0.6007 (6)	0.83451 (19)	0.0444 (18)	
H11A	0.5340	0.6748	0.8165	0.053*	
C3	0.2137 (8)	0.4159 (9)	0.8112 (2)	0.065 (2)	
H3A	0.1892	0.3324	0.8131	0.079*	
C15	0.5245 (7)	0.7010 (7)	0.9520 (2)	0.054 (2)	
C21	0.7237 (6)	0.5071 (7)	0.8481 (2)	0.052 (2)	
H21A	0.7125	0.5040	0.8765	0.063*	
H21B	0.7607	0.5874	0.8380	0.063*	
C13	0.5755 (7)	0.7402 (7)	0.8840 (2)	0.061 (2)	
H13A	0.6154	0.7890	0.8629	0.074*	
C17	0.4468 (7)	0.5657 (7)	0.9079 (2)	0.062 (2)	
H17A	0.3992	0.4968	0.9036	0.075*	
C16	0.4564 (8)	0.5992 (8)	0.9458 (2)	0.069 (2)	
H16A	0.4160	0.5518	0.9670	0.082*	
C20	0.8108 (7)	0.4022 (8)	0.8394 (2)	0.068 (2)	
H20A	0.8927	0.4119	0.8514	0.082*	
H20B	0.7765	0.3228	0.8516	0.082*	
C19	0.7104 (7)	0.3866 (7)	0.7802 (2)	0.060 (2)	
H19A	0.6722	0.3079	0.7913	0.072*	
H19B	0.7224	0.3851	0.7519	0.072*	
C14	0.5849 (8)	0.7745 (7)	0.9214 (3)	0.065 (2)	
H14A	0.6303	0.8448	0.9258	0.078*	
C10	0.2870 (8)	0.6678 (8)	0.8067 (2)	0.052 (2)	
C5	0.1623 (8)	0.6384 (11)	0.7935 (2)	0.067 (2)	
C9	0.3147 (8)	0.7970 (8)	0.8055 (2)	0.067 (2)	
H9A	0.3922	0.8200	0.8150	0.081*	
C4	0.1348 (8)	0.5093 (12)	0.7964 (3)	0.081 (3)	
H4A	0.0562	0.4877	0.7874	0.097*	
C8	0.2272 (11)	0.8900 (9)	0.7902 (3)	0.090 (3)	
H8A	0.2481	0.9744	0.7890	0.108*	
C6	0.0802 (9)	0.7374 (12)	0.7783 (3)	0.087 (3)	
H6A	0.0014	0.7179	0.7688	0.105*	
C7	0.1099 (11)	0.8595 (13)	0.7767 (3)	0.103 (4)	
H7A	0.0522	0.9227	0.7666	0.123*	
Br2	0.74914 (8)	0.46258 (9)	0.49468 (3)	0.0850 (3)	
O3	0.2734 (5)	0.5256 (5)	0.66728 (16)	0.0720 (16)	
H3B	0.3007	0.4595	0.6603	0.108*	
O4	0.3368 (6)	0.0926 (5)	0.7095 (2)	0.0871 (18)	
N2	0.4301 (5)	0.3323 (5)	0.67417 (17)	0.0502 (16)	
C22	0.4977 (7)	0.5507 (6)	0.67801 (19)	0.0404 (18)	
C32	0.5342 (6)	0.4245 (6)	0.6655 (2)	0.0438 (18)	
H32A	0.6056	0.3914	0.6824	0.053*	
C31	0.5940 (8)	0.6321 (7)	0.6884 (2)	0.0478 (19)	
C36	0.6813 (8)	0.4581 (8)	0.5472 (2)	0.059 (2)	
C33	0.5839 (7)	0.4356 (6)	0.6230 (2)	0.0449 (18)	
C38	0.5213 (7)	0.5069 (8)	0.5921 (2)	0.068 (2)	
H38A	0.4455	0.5487	0.5971	0.081*	
C40	0.3465 (9)	0.1249 (7)	0.6680 (3)	0.085 (3)	
H40A	0.2664	0.1627	0.6580	0.101*	
H40B	0.3616	0.0488	0.6559	0.101*	
C25	0.4293 (10)	0.7827 (8)	0.7022 (2)	0.067 (2)	
H25A	0.4052	0.8592	0.7106	0.081*	
C26	0.5586 (9)	0.7492 (8)	0.7015 (2)	0.057 (2)	
C39	0.4515 (7)	0.2154 (7)	0.6559 (2)	0.064 (2)	
H39A	0.5327	0.1767	0.6645	0.077*	
H39B	0.4538	0.2354	0.6273	0.077*	
C37	0.5690 (8)	0.5175 (8)	0.5539 (2)	0.072 (2)	
H37A	0.5252	0.5645	0.5331	0.086*	
C30	0.7248 (8)	0.6018 (7)	0.6862 (2)	0.056 (2)	
H30A	0.7505	0.5278	0.6766	0.067*	
C34	0.6971 (7)	0.3777 (7)	0.6150 (3)	0.066 (2)	
H34A	0.7427	0.3327	0.6358	0.079*	
C42	0.4178 (7)	0.2967 (7)	0.7178 (2)	0.065 (2)	
H42A	0.3979	0.3713	0.7302	0.078*	
H42B	0.4978	0.2607	0.7284	0.078*	
C29	0.8157 (8)	0.6801 (9)	0.6980 (2)	0.071 (2)	
H29A	0.9013	0.6567	0.6971	0.085*	
C27	0.6555 (11)	0.8284 (8)	0.7124 (2)	0.073 (3)	
H27A	0.6330	0.9055	0.7205	0.087*	
C23	0.3719 (8)	0.5925 (7)	0.6785 (2)	0.055 (2)	
C24	0.3385 (9)	0.7081 (8)	0.6913 (2)	0.068 (2)	
H24A	0.2534	0.7333	0.6923	0.082*	
C41	0.3144 (8)	0.2023 (7)	0.7271 (2)	0.073 (3)	
H41A	0.3080	0.1799	0.7556	0.088*	
H41B	0.2338	0.2406	0.7178	0.088*	
C28	0.7798 (11)	0.7937 (9)	0.7111 (2)	0.078 (3)	
H28A	0.8415	0.8458	0.7191	0.094*	
C35	0.7455 (7)	0.3843 (8)	0.5767 (3)	0.065 (2)	
H35A	0.8193	0.3400	0.5713	0.078*	
Br4	0.08240 (10)	0.62411 (8)	0.04967 (3)	0.0908 (4)	
O7	0.0940 (5)	−0.0686 (4)	0.03561 (15)	0.0605 (14)	
H7B	0.0253	−0.0336	0.0390	0.091*	
O8	−0.3346 (5)	−0.1270 (6)	0.07228 (16)	0.0684 (15)	
N4	−0.0974 (5)	−0.0084 (5)	0.07986 (16)	0.0445 (15)	
C75	0.0118 (7)	0.1946 (7)	0.0873 (2)	0.0496 (19)	
C74	−0.0022 (6)	0.0562 (6)	0.10232 (19)	0.0403 (18)	
H74A	−0.0308	0.0494	0.1300	0.048*	
C76	−0.0422 (7)	0.2842 (7)	0.1083 (3)	0.067 (2)	
H76A	−0.0919	0.2584	0.1306	0.081*	
C73	0.2143 (7)	−0.0146 (6)	0.1338 (2)	0.0459 (19)	
C65	0.1669 (7)	−0.0678 (6)	0.0680 (2)	0.0464 (19)	
C69	0.4202 (8)	−0.0709 (7)	0.1640 (3)	0.072 (3)	
H69A	0.5009	−0.1073	0.1623	0.087*	
C64	0.1265 (7)	−0.0126 (6)	0.1016 (2)	0.0445 (19)	
C81	−0.2165 (7)	0.0648 (7)	0.0718 (2)	0.061 (2)	
H81A	−0.1988	0.1443	0.0557	0.073*	
H81B	−0.2543	0.0829	0.0966	0.073*	
C78	0.0500 (8)	0.4470 (7)	0.0644 (3)	0.063 (2)	
C79	0.1017 (7)	0.3617 (7)	0.0423 (2)	0.067 (2)	
H79A	0.1494	0.3889	0.0197	0.080*	
C80	0.0834 (7)	0.2355 (7)	0.0534 (2)	0.065 (2)	
H80A	0.1188	0.1769	0.0384	0.078*	
C67	0.3703 (8)	−0.1266 (7)	0.0971 (3)	0.065 (2)	
H67A	0.4502	−0.1647	0.0955	0.078*	
C66	0.2890 (7)	−0.1238 (6)	0.0670 (2)	0.054 (2)	
H66A	0.3140	−0.1600	0.0447	0.065*	
C68	0.3353 (7)	−0.0718 (6)	0.1316 (3)	0.050 (2)	
C83	−0.2223 (8)	−0.1999 (7)	0.0798 (2)	0.063 (2)	
H83A	−0.1848	−0.2172	0.0548	0.075*	
H83B	−0.2424	−0.2799	0.0953	0.075*	
C84	−0.1271 (7)	−0.1319 (7)	0.1022 (2)	0.063 (2)	
H84A	−0.1621	−0.1201	0.1280	0.076*	
H84B	−0.0497	−0.1828	0.1063	0.076*	
C72	0.1825 (7)	0.0405 (7)	0.1682 (2)	0.056 (2)	
H72A	0.1027	0.0786	0.1702	0.068*	
C77	−0.0241 (8)	0.4116 (8)	0.0971 (3)	0.068 (2)	
H77A	−0.0613	0.4715	0.1113	0.082*	
C70	0.3881 (9)	−0.0193 (8)	0.1972 (3)	0.075 (3)	
H70A	0.4439	−0.0218	0.2183	0.091*	
C71	0.2653 (9)	0.0389 (8)	0.1982 (2)	0.071 (2)	
H71A	0.2416	0.0771	0.2202	0.085*	
C82	−0.3074 (7)	−0.0082 (8)	0.0506 (2)	0.063 (2)	
H82A	−0.3861	0.0405	0.0459	0.076*	
H82B	−0.2714	−0.0207	0.0250	0.076*	
Br3	0.65346 (8)	0.92999 (11)	0.45458 (3)	0.1049 (4)	
O5	−0.0484 (4)	0.9132 (5)	0.46306 (15)	0.0584 (14)	
H5A	−0.0136	0.9816	0.4595	0.088*	
O6	−0.1023 (6)	1.3581 (5)	0.42469 (16)	0.0695 (15)	
N3	0.0234 (5)	1.1195 (5)	0.41944 (16)	0.0446 (15)	
C52	0.0401 (6)	0.8411 (7)	0.3638 (2)	0.0454 (19)	
C54	0.2342 (7)	1.0095 (7)	0.4130 (2)	0.0499 (19)	
C44	−0.0357 (6)	0.8559 (7)	0.4299 (3)	0.051 (2)	
C47	−0.0175 (7)	0.7214 (7)	0.3659 (3)	0.055 (2)	
C45	−0.0902 (7)	0.7381 (8)	0.4316 (3)	0.065 (2)	
H45A	−0.1312	0.7034	0.4548	0.078*	
C61	−0.1706 (7)	1.2501 (8)	0.4179 (2)	0.066 (2)	
H61A	−0.2455	1.2767	0.4023	0.080*	
H61B	−0.1986	1.2058	0.4431	0.080*	
C51	0.1093 (7)	0.8830 (7)	0.3283 (2)	0.060 (2)	
H51A	0.1518	0.9588	0.3266	0.072*	
C59	0.3337 (7)	1.0740 (7)	0.3931 (2)	0.062 (2)	
H59A	0.3175	1.1313	0.3705	0.075*	
C63	0.0943 (7)	1.2306 (7)	0.4270 (2)	0.060 (2)	
H63A	0.1253	1.2747	0.4021	0.072*	
H63B	0.1671	1.2042	0.4436	0.072*	
C53	0.1006 (6)	1.0318 (6)	0.3973 (2)	0.0447 (18)	
H53A	0.1074	1.0715	0.3697	0.054*	
C55	0.2616 (7)	0.9222 (7)	0.4462 (2)	0.062 (2)	
H55A	0.1959	0.8793	0.4602	0.075*	
C43	0.0316 (6)	0.9085 (6)	0.3972 (2)	0.0431 (18)	
C56	0.3859 (8)	0.8993 (7)	0.4584 (2)	0.067 (2)	
H56A	0.4033	0.8401	0.4804	0.080*	
C46	−0.0846 (7)	0.6732 (8)	0.4002 (3)	0.072 (3)	
H46A	−0.1252	0.5965	0.4013	0.086*	
C50	0.1154 (8)	0.8161 (8)	0.2969 (3)	0.071 (2)	
H50A	0.1607	0.8475	0.2743	0.085*	
C48	−0.0106 (7)	0.6550 (7)	0.3323 (3)	0.070 (3)	
H48A	−0.0512	0.5783	0.3334	0.085*	
C60	−0.0906 (7)	1.1613 (7)	0.3964 (2)	0.060 (2)	
H60A	−0.1400	1.0887	0.3924	0.073*	
H60B	−0.0657	1.2037	0.3705	0.073*	
C62	0.0072 (8)	1.3189 (7)	0.4476 (2)	0.063 (2)	
H62A	−0.0191	1.2759	0.4732	0.076*	
H62B	0.0542	1.3926	0.4522	0.076*	
C58	0.4572 (8)	1.0534 (9)	0.4067 (3)	0.072 (3)	
H58A	0.5227	1.1010	0.3942	0.087*	
C57	0.4839 (7)	0.9628 (8)	0.4385 (3)	0.065 (2)	
C49	0.0547 (8)	0.7013 (9)	0.2983 (3)	0.080 (3)	
H49A	0.0580	0.6566	0.2766	0.096*	

### Atomic displacement parameters (Å^2^)

**Table:**

	*U*^11^	*U*^22^	*U*^33^	*U*^12^	*U*^13^	*U*^23^
Br1	0.1015 (8)	0.0790 (7)	0.0584 (6)	0.0112 (5)	−0.0019 (5)	−0.0239 (5)
O1	0.057 (4)	0.044 (3)	0.110 (5)	−0.004 (3)	0.003 (3)	−0.007 (3)
O2	0.048 (4)	0.093 (4)	0.066 (4)	0.003 (3)	0.010 (3)	−0.016 (3)
N1	0.038 (4)	0.050 (4)	0.045 (4)	−0.002 (3)	0.001 (3)	−0.009 (3)
C18	0.054 (5)	0.061 (5)	0.043 (5)	−0.011 (4)	−0.001 (4)	−0.011 (4)
C1	0.041 (5)	0.046 (5)	0.046 (5)	−0.001 (4)	0.008 (4)	−0.004 (4)
C12	0.052 (5)	0.054 (5)	0.040 (5)	−0.007 (4)	0.001 (4)	0.002 (4)
C2	0.055 (6)	0.061 (6)	0.062 (6)	−0.010 (5)	0.009 (4)	−0.017 (5)
C11	0.045 (5)	0.049 (5)	0.036 (5)	0.000 (4)	0.004 (4)	0.007 (4)
C3	0.042 (6)	0.082 (7)	0.075 (6)	−0.012 (5)	0.003 (5)	−0.021 (5)
C15	0.064 (6)	0.044 (5)	0.057 (6)	0.012 (4)	−0.009 (4)	−0.023 (4)
C21	0.045 (5)	0.060 (5)	0.054 (5)	−0.003 (4)	−0.002 (4)	−0.014 (4)
C13	0.080 (6)	0.052 (5)	0.050 (6)	−0.018 (5)	0.006 (5)	0.003 (4)
C17	0.076 (6)	0.056 (5)	0.056 (6)	−0.016 (4)	0.011 (5)	−0.010 (5)
C16	0.087 (6)	0.078 (6)	0.040 (5)	−0.027 (5)	0.020 (4)	0.001 (5)
C20	0.050 (5)	0.091 (7)	0.065 (6)	0.002 (5)	−0.004 (4)	−0.012 (5)
C19	0.059 (6)	0.072 (6)	0.053 (5)	−0.002 (5)	−0.008 (4)	−0.020 (4)
C14	0.079 (6)	0.047 (5)	0.067 (7)	−0.005 (4)	0.003 (5)	−0.002 (5)
C10	0.054 (6)	0.061 (6)	0.038 (5)	0.002 (5)	0.007 (4)	0.002 (4)
C5	0.044 (6)	0.105 (8)	0.048 (5)	0.014 (6)	0.006 (4)	−0.006 (5)
C9	0.072 (6)	0.063 (6)	0.062 (6)	−0.002 (5)	0.008 (5)	0.010 (5)
C4	0.047 (6)	0.121 (9)	0.081 (7)	−0.024 (7)	0.004 (5)	−0.036 (7)
C8	0.095 (8)	0.070 (7)	0.095 (8)	0.020 (7)	0.023 (7)	0.015 (6)
C6	0.066 (7)	0.120 (9)	0.069 (7)	0.027 (7)	−0.002 (5)	0.008 (7)
C7	0.079 (9)	0.131 (11)	0.082 (8)	0.044 (8)	0.007 (6)	0.037 (7)
Br2	0.0792 (7)	0.1115 (8)	0.0647 (7)	−0.0114 (6)	0.0219 (5)	−0.0139 (6)
O3	0.055 (4)	0.064 (4)	0.098 (5)	0.000 (3)	0.000 (3)	−0.017 (3)
O4	0.129 (5)	0.047 (4)	0.084 (5)	−0.013 (3)	0.025 (4)	−0.002 (3)
N2	0.068 (4)	0.041 (4)	0.044 (4)	−0.001 (3)	0.002 (3)	−0.014 (3)
C22	0.036 (5)	0.045 (5)	0.040 (4)	0.003 (4)	0.004 (3)	−0.005 (4)
C32	0.044 (5)	0.039 (4)	0.048 (5)	−0.002 (4)	−0.007 (4)	−0.004 (4)
C31	0.053 (6)	0.053 (5)	0.036 (5)	−0.004 (5)	−0.002 (4)	−0.003 (4)
C36	0.049 (5)	0.067 (6)	0.065 (6)	−0.008 (5)	0.017 (5)	−0.021 (5)
C33	0.047 (5)	0.043 (5)	0.045 (5)	−0.004 (4)	−0.004 (4)	−0.009 (4)
C38	0.058 (6)	0.089 (6)	0.055 (6)	0.028 (5)	0.006 (5)	−0.013 (5)
C40	0.098 (7)	0.048 (5)	0.112 (9)	−0.017 (5)	0.016 (6)	−0.027 (6)
C25	0.102 (8)	0.047 (6)	0.051 (6)	0.018 (6)	0.003 (5)	−0.001 (4)
C26	0.076 (7)	0.059 (6)	0.035 (5)	−0.003 (6)	−0.003 (4)	−0.004 (4)
C39	0.066 (6)	0.058 (5)	0.069 (6)	−0.010 (5)	0.004 (5)	−0.013 (5)
C37	0.070 (6)	0.089 (7)	0.051 (6)	0.025 (5)	−0.004 (5)	0.004 (5)
C30	0.056 (6)	0.069 (6)	0.042 (5)	−0.009 (5)	−0.002 (4)	−0.002 (4)
C34	0.049 (5)	0.074 (6)	0.071 (7)	0.015 (5)	0.006 (5)	0.000 (5)
C42	0.071 (6)	0.060 (5)	0.065 (6)	−0.011 (5)	0.007 (5)	−0.009 (4)
C29	0.061 (6)	0.086 (7)	0.061 (6)	−0.016 (6)	−0.009 (5)	0.012 (5)
C27	0.104 (8)	0.054 (6)	0.058 (6)	−0.003 (6)	−0.027 (6)	0.004 (4)
C23	0.065 (6)	0.042 (5)	0.055 (5)	−0.003 (5)	0.003 (4)	0.003 (4)
C24	0.083 (7)	0.045 (5)	0.076 (6)	0.012 (5)	0.016 (5)	−0.013 (5)
C41	0.090 (7)	0.058 (6)	0.070 (6)	−0.005 (5)	0.029 (5)	−0.004 (5)
C28	0.116 (9)	0.063 (7)	0.054 (6)	−0.031 (7)	−0.022 (6)	0.008 (5)
C35	0.048 (5)	0.075 (6)	0.073 (7)	0.013 (5)	0.003 (5)	−0.015 (5)
Br4	0.1229 (9)	0.0442 (5)	0.1052 (8)	−0.0055 (5)	−0.0244 (6)	−0.0072 (5)
O7	0.058 (3)	0.066 (4)	0.060 (4)	−0.001 (3)	0.008 (3)	−0.018 (3)
O8	0.038 (3)	0.085 (4)	0.080 (4)	−0.008 (3)	0.004 (3)	−0.003 (3)
N4	0.037 (4)	0.044 (4)	0.052 (4)	0.000 (3)	0.001 (3)	−0.006 (3)
C75	0.050 (5)	0.049 (5)	0.051 (5)	0.007 (4)	−0.002 (4)	−0.012 (4)
C74	0.044 (5)	0.042 (4)	0.035 (4)	−0.002 (4)	0.008 (3)	−0.006 (3)
C76	0.074 (6)	0.040 (5)	0.092 (7)	0.002 (4)	0.010 (5)	−0.027 (5)
C73	0.039 (5)	0.044 (5)	0.053 (5)	−0.011 (4)	0.005 (4)	0.000 (4)
C65	0.048 (5)	0.027 (4)	0.065 (6)	−0.006 (4)	0.014 (4)	−0.013 (4)
C69	0.047 (6)	0.055 (6)	0.108 (8)	−0.015 (4)	−0.015 (6)	0.021 (6)
C64	0.043 (5)	0.029 (4)	0.063 (5)	−0.011 (4)	0.008 (4)	−0.012 (4)
C81	0.049 (5)	0.053 (5)	0.082 (6)	0.001 (4)	−0.008 (4)	−0.013 (5)
C78	0.084 (7)	0.044 (5)	0.062 (6)	0.010 (5)	−0.025 (5)	−0.014 (5)
C79	0.081 (6)	0.047 (5)	0.071 (6)	0.000 (5)	0.001 (5)	−0.009 (5)
C80	0.083 (6)	0.042 (5)	0.071 (6)	0.013 (5)	0.001 (5)	−0.019 (5)
C67	0.044 (5)	0.046 (5)	0.102 (8)	−0.007 (4)	0.014 (5)	−0.001 (5)
C66	0.048 (5)	0.048 (5)	0.071 (6)	0.000 (4)	0.016 (5)	−0.027 (4)
C68	0.037 (5)	0.028 (4)	0.084 (7)	−0.004 (4)	0.005 (5)	0.003 (4)
C83	0.069 (6)	0.060 (5)	0.057 (6)	−0.023 (5)	−0.002 (5)	0.006 (4)
C84	0.056 (5)	0.055 (5)	0.075 (6)	0.002 (4)	0.005 (5)	0.007 (5)
C72	0.051 (5)	0.069 (6)	0.051 (5)	−0.007 (4)	−0.004 (4)	−0.013 (4)
C77	0.079 (6)	0.055 (6)	0.078 (7)	0.006 (5)	−0.001 (5)	−0.037 (5)
C70	0.065 (7)	0.084 (7)	0.075 (7)	−0.012 (5)	−0.020 (5)	−0.003 (6)
C71	0.068 (6)	0.090 (7)	0.055 (6)	−0.007 (5)	−0.007 (5)	−0.013 (5)
C82	0.049 (5)	0.070 (6)	0.069 (6)	0.011 (5)	−0.005 (4)	0.001 (5)
Br3	0.0455 (6)	0.1376 (10)	0.1382 (10)	0.0112 (6)	−0.0114 (6)	−0.0444 (8)
O5	0.051 (3)	0.063 (3)	0.057 (4)	−0.004 (3)	0.012 (3)	0.008 (3)
O6	0.082 (4)	0.052 (4)	0.076 (4)	0.007 (3)	−0.002 (3)	−0.016 (3)
N3	0.045 (4)	0.035 (3)	0.051 (4)	0.007 (3)	−0.004 (3)	0.000 (3)
C52	0.024 (4)	0.041 (5)	0.072 (6)	0.001 (4)	0.003 (4)	−0.010 (4)
C54	0.050 (5)	0.051 (5)	0.050 (5)	−0.005 (4)	0.004 (4)	−0.010 (4)
C44	0.032 (5)	0.044 (5)	0.076 (7)	0.002 (4)	0.006 (4)	0.000 (5)
C47	0.029 (5)	0.044 (5)	0.094 (7)	0.004 (4)	−0.007 (4)	−0.019 (5)
C45	0.046 (5)	0.063 (6)	0.082 (7)	−0.007 (5)	0.020 (5)	0.006 (5)
C61	0.058 (5)	0.070 (6)	0.069 (6)	0.019 (5)	−0.012 (4)	−0.004 (5)
C51	0.067 (6)	0.044 (5)	0.068 (6)	−0.002 (4)	0.009 (5)	−0.006 (5)
C59	0.037 (5)	0.074 (6)	0.077 (6)	−0.005 (5)	0.014 (5)	−0.016 (5)
C63	0.061 (5)	0.060 (5)	0.060 (6)	−0.008 (5)	−0.006 (4)	−0.011 (4)
C53	0.032 (4)	0.050 (5)	0.051 (5)	−0.005 (4)	0.004 (4)	−0.001 (4)
C55	0.042 (5)	0.070 (6)	0.072 (6)	−0.001 (4)	0.008 (4)	0.002 (5)
C43	0.031 (4)	0.036 (4)	0.059 (5)	0.001 (4)	0.000 (4)	0.003 (4)
C56	0.060 (6)	0.071 (6)	0.067 (6)	0.004 (5)	0.001 (5)	−0.003 (5)
C46	0.044 (5)	0.048 (5)	0.120 (9)	−0.003 (4)	0.009 (6)	0.001 (6)
C50	0.073 (6)	0.070 (6)	0.073 (7)	0.001 (5)	0.007 (5)	−0.025 (5)
C48	0.044 (5)	0.049 (5)	0.125 (9)	−0.005 (4)	−0.010 (6)	−0.032 (6)
C60	0.067 (6)	0.044 (5)	0.070 (6)	0.012 (4)	−0.005 (5)	−0.005 (4)
C62	0.069 (6)	0.055 (5)	0.071 (6)	−0.002 (5)	−0.002 (5)	−0.022 (5)
C58	0.049 (6)	0.090 (7)	0.079 (7)	−0.016 (5)	0.021 (5)	−0.019 (6)
C57	0.036 (5)	0.074 (6)	0.090 (7)	0.002 (5)	0.009 (5)	−0.032 (6)
C49	0.059 (6)	0.090 (8)	0.097 (8)	0.005 (6)	0.000 (6)	−0.037 (6)

### Geometric parameters (Å, º)

**Table:**

Br1—C15	1.911 (7)	Br4—C78	1.932 (8)
O1—C2	1.354 (9)	O7—C65	1.349 (8)
O1—H1A	0.8200	O7—H7B	0.8200
O2—C19	1.406 (8)	O8—C83	1.406 (8)
O2—C20	1.429 (8)	O8—C82	1.416 (8)
N1—C18	1.474 (8)	N4—C81	1.470 (8)
N1—C21	1.477 (8)	N4—C84	1.472 (8)
N1—C11	1.488 (8)	N4—C74	1.501 (8)
C18—C19	1.500 (9)	C75—C76	1.379 (9)
C18—H18A	0.9700	C75—C80	1.396 (9)
C18—H18B	0.9700	C75—C74	1.510 (9)
C1—C2	1.382 (9)	C74—C64	1.526 (9)
C1—C10	1.408 (9)	C74—H74A	0.9800
C1—C11	1.537 (9)	C76—C77	1.383 (10)
C12—C13	1.375 (9)	C76—H76A	0.9300
C12—C17	1.395 (9)	C73—C68	1.405 (9)
C12—C11	1.526 (9)	C73—C72	1.412 (9)
C2—C3	1.421 (10)	C73—C64	1.437 (9)
C11—H11A	0.9800	C65—C66	1.406 (9)
C3—C4	1.333 (11)	C65—C64	1.411 (9)
C3—H3A	0.9300	C69—C70	1.357 (11)
C15—C16	1.358 (9)	C69—C68	1.425 (10)
C15—C14	1.378 (10)	C69—H69A	0.9300
C21—C20	1.490 (9)	C81—C82	1.498 (9)
C21—H21A	0.9700	C81—H81A	0.9700
C21—H21B	0.9700	C81—H81B	0.9700
C13—C14	1.376 (10)	C78—C79	1.358 (10)
C13—H13A	0.9300	C78—C77	1.367 (10)
C17—C16	1.387 (10)	C79—C80	1.370 (9)
C17—H17A	0.9300	C79—H79A	0.9300
C16—H16A	0.9300	C80—H80A	0.9300
C20—H20A	0.9700	C67—C66	1.336 (10)
C20—H20B	0.9700	C67—C68	1.421 (10)
C19—H19A	0.9700	C67—H67A	0.9300
C19—H19B	0.9700	C66—H66A	0.9300
C14—H14A	0.9300	C83—C84	1.519 (9)
C10—C9	1.415 (10)	C83—H83A	0.9700
C10—C5	1.445 (10)	C83—H83B	0.9700
C5—C6	1.398 (11)	C84—H84A	0.9700
C5—C4	1.408 (11)	C84—H84B	0.9700
C9—C8	1.391 (11)	C72—C71	1.346 (9)
C9—H9A	0.9300	C72—H72A	0.9300
C4—H4A	0.9300	C77—H77A	0.9300
C8—C7	1.382 (13)	C70—C71	1.422 (11)
C8—H8A	0.9300	C70—H70A	0.9300
C6—C7	1.345 (13)	C71—H71A	0.9300
C6—H6A	0.9300	C82—H82A	0.9700
C7—H7A	0.9300	C82—H82B	0.9700
Br2—C36	1.912 (8)	Br3—C57	1.882 (8)
O3—C23	1.359 (8)	O5—C44	1.357 (8)
O3—H3B	0.8200	O5—H5A	0.8200
O4—C41	1.402 (8)	O6—C61	1.421 (8)
O4—C40	1.409 (9)	O6—C62	1.421 (8)
N2—C39	1.480 (8)	N3—C60	1.466 (8)
N2—C42	1.485 (8)	N3—C63	1.470 (8)
N2—C32	1.491 (8)	N3—C53	1.499 (8)
C22—C23	1.387 (9)	C52—C47	1.422 (9)
C22—C31	1.429 (9)	C52—C43	1.425 (9)
C22—C32	1.510 (8)	C52—C51	1.427 (9)
C32—C33	1.521 (9)	C54—C59	1.387 (9)
C32—H32A	0.9800	C54—C55	1.395 (9)
C31—C30	1.409 (9)	C54—C53	1.509 (9)
C31—C26	1.425 (10)	C44—C43	1.376 (9)
C36—C37	1.360 (10)	C44—C45	1.391 (10)
C36—C35	1.364 (10)	C47—C46	1.402 (11)
C33—C34	1.372 (9)	C47—C48	1.422 (10)
C33—C38	1.377 (9)	C45—C46	1.350 (10)
C38—C37	1.378 (10)	C45—H45A	0.9300
C38—H38A	0.9300	C61—C60	1.511 (9)
C40—C39	1.503 (10)	C61—H61A	0.9700
C40—H40A	0.9700	C61—H61B	0.9700
C40—H40B	0.9700	C51—C50	1.363 (9)
C25—C24	1.344 (10)	C51—H51A	0.9300
C25—C26	1.399 (10)	C59—C58	1.386 (10)
C25—H25A	0.9300	C59—H59A	0.9300
C26—C27	1.423 (10)	C63—C62	1.533 (9)
C39—H39A	0.9700	C63—H63A	0.9700
C39—H39B	0.9700	C63—H63B	0.9700
C37—H37A	0.9300	C53—C43	1.522 (9)
C30—C29	1.382 (10)	C53—H53A	0.9800
C30—H30A	0.9300	C55—C56	1.382 (10)
C34—C35	1.386 (10)	C55—H55A	0.9300
C34—H34A	0.9300	C56—C57	1.369 (10)
C42—C41	1.503 (9)	C56—H56A	0.9300
C42—H42A	0.9700	C46—H46A	0.9300
C42—H42B	0.9700	C50—C49	1.392 (10)
C29—C28	1.393 (11)	C50—H50A	0.9300
C29—H29A	0.9300	C48—C49	1.380 (11)
C27—C28	1.352 (11)	C48—H48A	0.9300
C27—H27A	0.9300	C60—H60A	0.9700
C23—C24	1.401 (9)	C60—H60B	0.9700
C24—H24A	0.9300	C62—H62A	0.9700
C41—H41A	0.9700	C62—H62B	0.9700
C41—H41B	0.9700	C58—C57	1.380 (11)
C28—H28A	0.9300	C58—H58A	0.9300
C35—H35A	0.9300	C49—H49A	0.9300
			
C2—O1—H1A	109.5	C65—O7—H7B	109.5
C19—O2—C20	109.3 (5)	C83—O8—C82	110.6 (5)
C18—N1—C21	107.3 (5)	C81—N4—C84	108.8 (5)
C18—N1—C11	109.0 (5)	C81—N4—C74	113.5 (5)
C21—N1—C11	114.4 (5)	C84—N4—C74	109.7 (5)
N1—C18—C19	110.5 (6)	C76—C75—C80	118.4 (7)
N1—C18—H18A	109.5	C76—C75—C74	119.8 (7)
C19—C18—H18A	109.5	C80—C75—C74	121.8 (6)
N1—C18—H18B	109.5	N4—C74—C75	113.9 (5)
C19—C18—H18B	109.5	N4—C74—C64	109.4 (5)
H18A—C18—H18B	108.1	C75—C74—C64	110.2 (5)
C2—C1—C10	119.7 (7)	N4—C74—H74A	107.7
C2—C1—C11	119.9 (7)	C75—C74—H74A	107.7
C10—C1—C11	120.4 (7)	C64—C74—H74A	107.7
C13—C12—C17	118.5 (7)	C75—C76—C77	121.4 (8)
C13—C12—C11	120.0 (7)	C75—C76—H76A	119.3
C17—C12—C11	121.5 (7)	C77—C76—H76A	119.3
O1—C2—C1	125.2 (7)	C68—C73—C72	118.2 (7)
O1—C2—C3	114.1 (7)	C68—C73—C64	120.3 (7)
C1—C2—C3	120.7 (8)	C72—C73—C64	121.6 (7)
N1—C11—C12	112.0 (5)	O7—C65—C66	116.9 (7)
N1—C11—C1	111.2 (5)	O7—C65—C64	123.1 (7)
C12—C11—C1	112.8 (5)	C66—C65—C64	120.0 (8)
N1—C11—H11A	106.8	C70—C69—C68	122.8 (9)
C12—C11—H11A	106.8	C70—C69—H69A	118.6
C1—C11—H11A	106.8	C68—C69—H69A	118.6
C4—C3—C2	118.9 (8)	C65—C64—C73	117.8 (7)
C4—C3—H3A	120.6	C65—C64—C74	121.1 (7)
C2—C3—H3A	120.6	C73—C64—C74	120.9 (6)
C16—C15—C14	121.7 (7)	N4—C81—C82	110.0 (6)
C16—C15—Br1	121.0 (7)	N4—C81—H81A	109.7
C14—C15—Br1	117.2 (6)	C82—C81—H81A	109.7
N1—C21—C20	110.1 (6)	N4—C81—H81B	109.7
N1—C21—H21A	109.6	C82—C81—H81B	109.7
C20—C21—H21A	109.6	H81A—C81—H81B	108.2
N1—C21—H21B	109.6	C79—C78—C77	122.1 (8)
C20—C21—H21B	109.6	C79—C78—Br4	119.3 (7)
H21A—C21—H21B	108.1	C77—C78—Br4	118.6 (6)
C12—C13—C14	122.6 (7)	C78—C79—C80	119.7 (8)
C12—C13—H13A	118.7	C78—C79—H79A	120.1
C14—C13—H13A	118.7	C80—C79—H79A	120.1
C16—C17—C12	119.3 (7)	C79—C80—C75	120.2 (7)
C16—C17—H17A	120.4	C79—C80—H80A	119.9
C12—C17—H17A	120.4	C75—C80—H80A	119.9
C15—C16—C17	120.4 (7)	C66—C67—C68	120.4 (8)
C15—C16—H16A	119.8	C66—C67—H67A	119.8
C17—C16—H16A	119.8	C68—C67—H67A	119.8
O2—C20—C21	113.3 (6)	C67—C66—C65	122.1 (8)
O2—C20—H20A	108.9	C67—C66—H66A	118.9
C21—C20—H20A	108.9	C65—C66—H66A	118.9
O2—C20—H20B	108.9	C73—C68—C67	119.3 (8)
C21—C20—H20B	108.9	C73—C68—C69	118.6 (8)
H20A—C20—H20B	107.7	C67—C68—C69	122.0 (8)
O2—C19—C18	111.2 (6)	O8—C83—C84	111.0 (6)
O2—C19—H19A	109.4	O8—C83—H83A	109.4
C18—C19—H19A	109.4	C84—C83—H83A	109.4
O2—C19—H19B	109.4	O8—C83—H83B	109.4
C18—C19—H19B	109.4	C84—C83—H83B	109.4
H19A—C19—H19B	108.0	H83A—C83—H83B	108.0
C13—C14—C15	117.6 (7)	N4—C84—C83	110.7 (6)
C13—C14—H14A	121.2	N4—C84—H84A	109.5
C15—C14—H14A	121.2	C83—C84—H84A	109.5
C1—C10—C9	122.8 (8)	N4—C84—H84B	109.5
C1—C10—C5	120.2 (8)	C83—C84—H84B	109.5
C9—C10—C5	116.9 (8)	H84A—C84—H84B	108.1
C6—C5—C4	125.2 (10)	C71—C72—C73	121.3 (8)
C6—C5—C10	118.8 (10)	C71—C72—H72A	119.3
C4—C5—C10	116.0 (8)	C73—C72—H72A	119.3
C8—C9—C10	120.6 (9)	C78—C77—C76	118.1 (7)
C8—C9—H9A	119.7	C78—C77—H77A	121.0
C10—C9—H9A	119.7	C76—C77—H77A	121.0
C3—C4—C5	124.5 (9)	C69—C70—C71	117.0 (8)
C3—C4—H4A	117.7	C69—C70—H70A	121.5
C5—C4—H4A	117.7	C71—C70—H70A	121.5
C7—C8—C9	121.3 (10)	C72—C71—C70	122.0 (8)
C7—C8—H8A	119.3	C72—C71—H71A	119.0
C9—C8—H8A	119.3	C70—C71—H71A	119.0
C7—C6—C5	123.0 (10)	O8—C82—C81	112.4 (6)
C7—C6—H6A	118.5	O8—C82—H82A	109.1
C5—C6—H6A	118.5	C81—C82—H82A	109.1
C6—C7—C8	119.2 (10)	O8—C82—H82B	109.1
C6—C7—H7A	120.4	C81—C82—H82B	109.1
C8—C7—H7A	120.4	H82A—C82—H82B	107.9
C23—O3—H3B	109.5	C44—O5—H5A	109.5
C41—O4—C40	109.2 (6)	C61—O6—C62	109.1 (5)
C39—N2—C42	108.1 (6)	C60—N3—C63	109.1 (5)
C39—N2—C32	113.2 (5)	C60—N3—C53	108.9 (5)
C42—N2—C32	109.6 (5)	C63—N3—C53	112.7 (5)
C23—C22—C31	118.4 (7)	C47—C52—C43	119.2 (7)
C23—C22—C32	121.6 (6)	C47—C52—C51	116.7 (7)
C31—C22—C32	120.0 (6)	C43—C52—C51	124.0 (7)
N2—C32—C22	111.3 (5)	C59—C54—C55	118.7 (7)
N2—C32—C33	113.9 (5)	C59—C54—C53	119.4 (7)
C22—C32—C33	112.7 (5)	C55—C54—C53	121.9 (7)
N2—C32—H32A	106.1	O5—C44—C43	122.2 (7)
C22—C32—H32A	106.1	O5—C44—C45	116.0 (7)
C33—C32—H32A	106.1	C43—C44—C45	121.8 (8)
C30—C31—C26	117.8 (7)	C46—C47—C52	119.7 (8)
C30—C31—C22	122.6 (7)	C46—C47—C48	121.1 (8)
C26—C31—C22	119.6 (7)	C52—C47—C48	119.2 (8)
C37—C36—C35	122.1 (8)	C46—C45—C44	121.0 (8)
C37—C36—Br2	121.0 (7)	C46—C45—H45A	119.5
C35—C36—Br2	116.7 (6)	C44—C45—H45A	119.5
C34—C33—C38	118.1 (7)	O6—C61—C60	112.0 (6)
C34—C33—C32	119.7 (7)	O6—C61—H61A	109.2
C38—C33—C32	122.1 (7)	C60—C61—H61A	109.2
C33—C38—C37	121.2 (7)	O6—C61—H61B	109.2
C33—C38—H38A	119.4	C60—C61—H61B	109.2
C37—C38—H38A	119.4	H61A—C61—H61B	107.9
O4—C40—C39	112.8 (7)	C50—C51—C52	122.5 (8)
O4—C40—H40A	109.0	C50—C51—H51A	118.8
C39—C40—H40A	109.0	C52—C51—H51A	118.8
O4—C40—H40B	109.0	C58—C59—C54	120.2 (8)
C39—C40—H40B	109.0	C58—C59—H59A	119.9
H40A—C40—H40B	107.8	C54—C59—H59A	119.9
C24—C25—C26	122.2 (8)	N3—C63—C62	109.7 (6)
C24—C25—H25A	118.9	N3—C63—H63A	109.7
C26—C25—H25A	118.9	C62—C63—H63A	109.7
C25—C26—C27	122.7 (9)	N3—C63—H63B	109.7
C25—C26—C31	118.3 (8)	C62—C63—H63B	109.7
C27—C26—C31	119.0 (8)	H63A—C63—H63B	108.2
N2—C39—C40	109.8 (6)	N3—C53—C54	112.9 (6)
N2—C39—H39A	109.7	N3—C53—C43	110.1 (5)
C40—C39—H39A	109.7	C54—C53—C43	111.6 (5)
N2—C39—H39B	109.7	N3—C53—H53A	107.3
C40—C39—H39B	109.7	C54—C53—H53A	107.3
H39A—C39—H39B	108.2	C43—C53—H53A	107.3
C36—C37—C38	118.8 (8)	C56—C55—C54	120.3 (7)
C36—C37—H37A	120.6	C56—C55—H55A	119.9
C38—C37—H37A	120.6	C54—C55—H55A	119.9
C29—C30—C31	121.3 (8)	C44—C43—C52	118.1 (7)
C29—C30—H30A	119.4	C44—C43—C53	120.9 (7)
C31—C30—H30A	119.4	C52—C43—C53	120.9 (6)
C33—C34—C35	121.7 (8)	C57—C56—C55	120.8 (8)
C33—C34—H34A	119.1	C57—C56—H56A	119.6
C35—C34—H34A	119.1	C55—C56—H56A	119.6
N2—C42—C41	110.3 (6)	C45—C46—C47	120.0 (8)
N2—C42—H42A	109.6	C45—C46—H46A	120.0
C41—C42—H42A	109.6	C47—C46—H46A	120.0
N2—C42—H42B	109.6	C51—C50—C49	120.9 (8)
C41—C42—H42B	109.6	C51—C50—H50A	119.5
H42A—C42—H42B	108.1	C49—C50—H50A	119.5
C30—C29—C28	120.4 (8)	C49—C48—C47	121.8 (8)
C30—C29—H29A	119.8	C49—C48—H48A	119.1
C28—C29—H29A	119.8	C47—C48—H48A	119.1
C28—C27—C26	121.3 (9)	N3—C60—C61	110.2 (6)
C28—C27—H27A	119.3	N3—C60—H60A	109.6
C26—C27—H27A	119.3	C61—C60—H60A	109.6
O3—C23—C22	123.3 (7)	N3—C60—H60B	109.6
O3—C23—C24	115.5 (8)	C61—C60—H60B	109.6
C22—C23—C24	121.2 (8)	H60A—C60—H60B	108.1
C25—C24—C23	120.1 (8)	O6—C62—C63	111.8 (6)
C25—C24—H24A	119.9	O6—C62—H62A	109.3
C23—C24—H24A	119.9	C63—C62—H62A	109.3
O4—C41—C42	112.4 (6)	O6—C62—H62B	109.3
O4—C41—H41A	109.1	C63—C62—H62B	109.3
C42—C41—H41A	109.1	H62A—C62—H62B	107.9
O4—C41—H41B	109.1	C57—C58—C59	120.5 (8)
C42—C41—H41B	109.1	C57—C58—H58A	119.7
H41A—C41—H41B	107.9	C59—C58—H58A	119.7
C27—C28—C29	120.2 (9)	C56—C57—C58	119.3 (8)
C27—C28—H28A	119.9	C56—C57—Br3	120.9 (8)
C29—C28—H28A	119.9	C58—C57—Br3	119.7 (7)
C36—C35—C34	117.9 (7)	C48—C49—C50	118.8 (9)
C36—C35—H35A	121.1	C48—C49—H49A	120.6
C34—C35—H35A	121.1	C50—C49—H49A	120.6

### Hydrogen-bond geometry (Å, º)

**Table:**

*D*—H···*A*	*D*—H	H···*A*	*D*···*A*	*D*—H···*A*
O1—H1*A*···N1	0.82	1.92	2.600 (7)	139
O3—H3*B*···N2	0.82	1.92	2.601 (7)	140
O5—H5*A*···N3	0.82	1.91	2.612 (7)	142
O7—H7*B*···N4	0.82	1.93	2.620 (7)	142
C25—H25*A*···O4^i^	0.93	2.58	3.473 (10)	162
C46—H46*A*···O6^ii^	0.93	2.57	3.371 (10)	144
C55—H55*A*···O5	0.93	2.59	3.305 (9)	134
C67—H67*A*···O8^iii^	0.93	2.41	3.208 (9)	143

Symmetry codes: (i) *x*, *y*+1, *z*; (ii) *x*, *y*−1, *z*; (iii) *x*+1, *y*, *z*.
